# Elevated Cardiac Troponin to Detect Acute Cellular Rejection After Cardiac Transplantation: A Systematic Review and Meta-Analysis

**DOI:** 10.3389/ti.2022.10362

**Published:** 2022-06-08

**Authors:** Zhengyang Liu, Luke A. Perry, Jahan C. Penny-Dimri, Michael Handscombe, Isabella Overmars, Mark Plummer, Reny Segal, Julian A. Smith

**Affiliations:** ^1^ Department of Anaesthesia, Royal Melbourne Hospital, Parkville, VIC, Australia; ^2^ Department of Surgery, School of Clinical Sciences at Monash Health, Monash University, Clayton, VIC, Australia; ^3^ Infection and Immunity Theme, Murdoch Children’s Research Institute, Parkville, VIC, Australia; ^4^ Department of Intensive Care Medicine, Royal Melbourne Hospital, Parkville, VIC, Australia; ^5^ Department of Medicine, University of Melbourne, Parkville, VIC, Australia

**Keywords:** heart transplantation, meta-analysis, systematic review, cardiac troponin, acute cellular rejection

## Abstract

Cardiac troponin is well known as a highly specific marker of cardiomyocyte damage, and has significant diagnostic accuracy in many cardiac conditions. However, the value of elevated recipient troponin in diagnosing adverse outcomes in heart transplant recipients is uncertain. We searched MEDLINE (Ovid), Embase (Ovid), and the Cochrane Library from inception until December 2020. We generated summary sensitivity, specificity, and Bayesian areas under the curve (BAUC) using bivariate Bayesian modelling, and standardised mean differences (SMDs) to quantify the diagnostic relationship of recipient troponin and adverse outcomes following cardiac transplant. We included 27 studies with 1,684 cardiac transplant recipients. Patients with acute rejection had a statistically significant late elevation in standardised troponin measurements taken at least 1 month postoperatively (SMD 0.98, 95% CI 0.33–1.64). However, pooled diagnostic accuracy was poor (sensitivity 0.414, 95% CrI 0.174–0.696; specificity 0.785, 95% CrI 0.567–0.912; BAUC 0.607, 95% CrI 0.469–0.723). In summary, late troponin elevation in heart transplant recipients is associated with acute cellular rejection in adults, but its stand-alone diagnostic accuracy is poor. Further research is needed to assess its performance in predictive modelling of adverse outcomes following cardiac transplant.

**Systematic Review Registration:** identifier CRD42021227861

## GRAPHICAL ABSTRACT

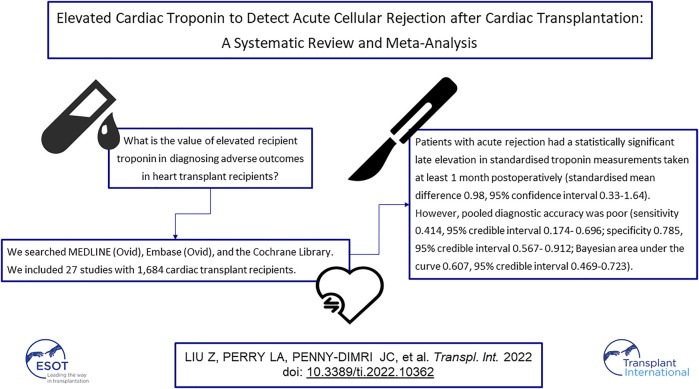

## Introduction

The endomyocardial biopsy (EMB) has remained the gold standard for detecting acute allograft rejection after cardiac transplant since its introduction in the early 1970s (1). However, this diagnostic test is invasive, can be poorly concordant amongst grading pathologists (2), and repeat procedures are associated with small but significant risks of complications including tricuspid regurgitation, cardiac tamponade, arrhythmias, and haemorrhage (3–5).

In light of these challenges, various biomarkers have been explored as diagnostic alternatives to EMB, contributing to an emerging sphere of multidisciplinary interest in the predictive (both diagnostic and prognostic) ability of routine serum biomarkers for adverse outcomes in a variety of conditions (6–13). In particular, cardiac troponin, a sensitive and specific marker of myocardial injury, is of broad prognostic significance across a range of cardiovascular diseases (14, 15). Although most classically elevated in the context of acute coronary syndromes, elevated troponin levels are also associated with a range of other cardiac and non-cardiac conditions including atrial fibrillation, congestive cardiac failure, myocarditis, myocardial contusion, pulmonary embolism, sepsis, renal failure, and hypovolaemia (16). Both donor and recipient troponin have been associated with adverse outcomes following cardiac transplant (17, 18). We have previously found that troponin elevations in cardiac transplant recipients may be prognostic for primary graft failure, adverse cardiac events, coronary artery disease, and long-term mortality, but its prognostic value in the context of acute rejection up to 1 year after transplant was uncertain (19). Donor troponin elevations though, were not associated with increased 30-day, 1-year, or long-term mortality post cardiac transplant despite increasing the risk of graft rejection at 1 year (but not at 30 days) (20).

However, the diagnostic utility of elevated cardiac troponin is controversial, and this biomarker has yet to be routinely integrated into the diagnostic pathway for acute allograft rejection or recommended by international guidelines (21, 22). Hence, we conducted this systematic review and meta-analysis of elevated cardiac troponin in diagnosing acute allograft rejection in heart transplant recipients.

## Methods

### Study Design and Registration

This systematic review and meta-analysis evaluated study level data, and was reported in compliance with the Meta-analysis Of Observational Studies in Epidemiology (MOOSE) guidelines (23). Protocol details were prospectively registered on PROSPERO (CRD42021227861) and there were no major protocol deviations.

### Eligibility Criteria

We included all original research studies which reported the diagnostic accuracy of elevated recipient troponin to detect adverse outcomes in heart transplant recipients. We excluded non-human studies, abstracts and conference presentations, case reports and series, editorials and expert opinions, review articles, and studies with incompletely reported data.

### Search Strategy

We searched MEDLINE (Ovid), Embase (Ovid), and the Cochrane Library from inception to December 2020. Our search strategy included a comprehensive set of search terms for troponin and cardiac transplantation (Supplementary Material) (24). We placed no restrictions on language or publication period.

### Study Selection

Two authors (ZL and MH) independently screened titles and abstracts of each search result for potentially relevant studies. The same two authors assessed full texts of shortlisted studies against eligibility criteria, with a third author (LAP) adjudicating any disagreements. We reviewed the reference and citation lists of included studies for further potentially relevant studies.

### Data Extraction and Management

Two authors (ZL and LAP) independently extracted data from included studies using standardised spreadsheets. We recorded the following, where reported and applicable: study design, population baseline characteristics including comorbidities, operative details, troponin type and measurement details, troponin threshold, definitional threshold of significant rejection by the International Society for Heart and Lung Transplantation (ISHLT) acute cellular rejection grade (25), outcomes, and diagnostic performance measures. Where studies reported dichotomous measures of diagnostic performance, we standardised reported data in confusion matrices and calculated sensitivity and specificity values; where studies reported continuous measures of effect, we standardised data reported as mean and standard deviation and calculated standardised mean differences (SMDs) (26).

### Assessment of Methodological Quality and Risk of Bias

Two authors (ZL and LAP) independently assessed the methodological quality of included studies using a modified version of the Quality Assessment of Diagnostic Accuracy Studies 2 (QUADAS-2) tool (27), with discrepancies resolved through discussion with a third author (MH). For this study, we expanded the grading of overall risk of bias to three categories (low, unclear, and high risk) from 2 categories (low risk and at risk), for greater consistency with the domain level risk of bias reporting (also low, unclear, and high risk) (28).

### Statistical Analysis and Data Synthesis

A detailed description of the statistical analysis is provided in the Supplementary Material. Anticipating significant between study variation in included studies, we pre-specified the use of random-effects models in all meta-analyses performed. Where studies reported continuous effect measures, we tabulated SMDs and associated confidence intervals (CIs) of recipient troponin measurements between acute cellular rejection and non-rejection groups, and used random effects inverse variance modelling to generate pooled SMDs. Where studies reported dichotomous effect measures and used receiver operating characteristic (ROC) analysis we noted optimised cut-off values, areas under the ROC curve (AUCs), sensitivities, specificities, and associated 95% CIs. From these, we calculated true positive, false positive, false negative, and true negative rates, and generated Bayesian Summary ROC (BSROC) curves and summary sensitivity, specificity, and Bayesian AUC (BAUC) statistics with 95% credible intervals (CrI) using a bivariate Bayesian modelling approach (29).

We estimated statistical heterogeneity using the I^2^ statistic for each meta-analysis. Where reporting of pre-specified covariates was sufficient across included studies, we used meta-regressions to explore possible sources of heterogeneity.

Where there were more than 10 included studies, we formally assessed publication bias with visual inspection of funnel plot skew and a regression test for funnel plot asymmetry (30). All analyses and figures were generated using Review Manager (RevMan) 5.4 (31) and the R statistical packages “metafor” (32) and “bamdit” (33).

## Results

### Search Results

**FIGURE 1:**
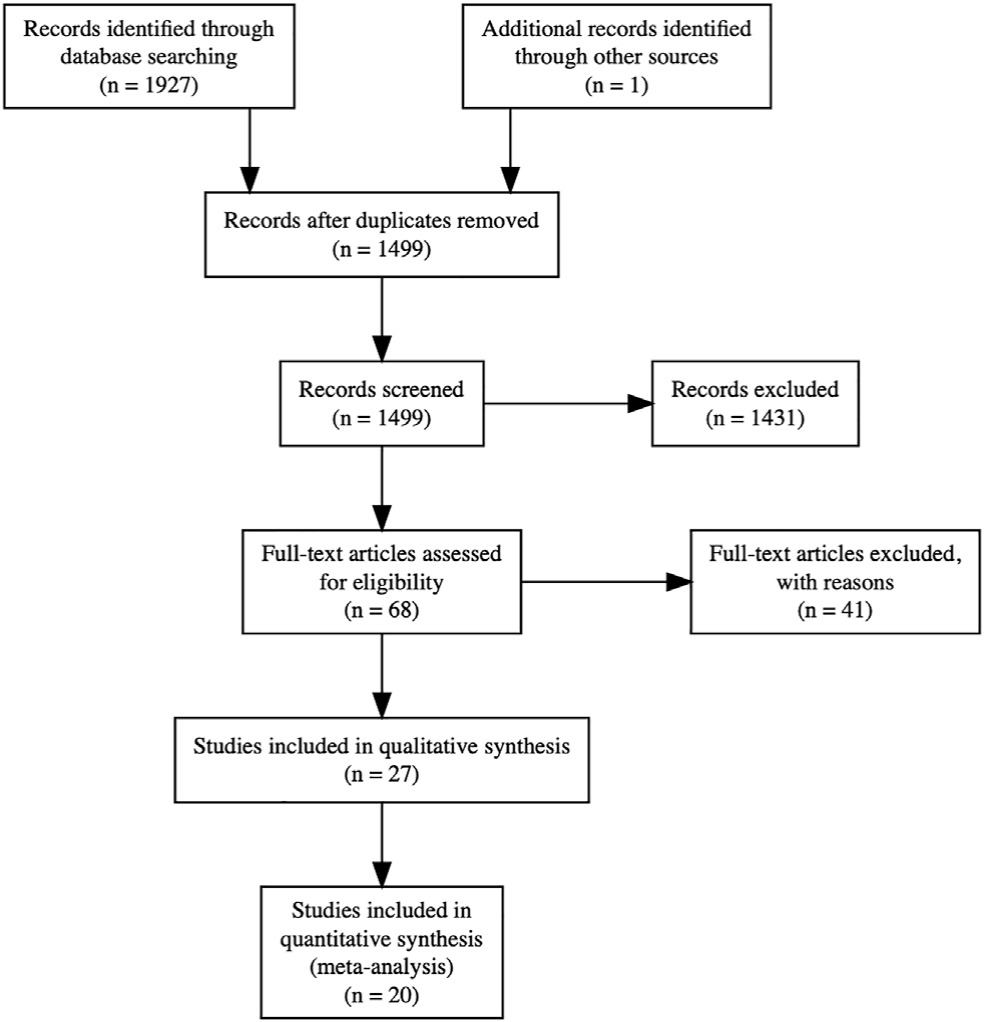
Preferred Reporting Items for Systematic Reviews and Meta-Analyses (PRISMA) flow diagram. Full text articles were excluded for the following reasons: 19 due to incorrect exposure measurement (donor troponin rather than recipient troponin), 15 due to incorrect study design (prognostic rather than diagnostic), 4 due to lack of troponin reporting, and 3 due to incorrect outcome measurement. Twenty studies were included in quantitative syntheses: for acute cellular rejection in adults with no exclusion of measurements from the early postoperative period, 8 studies were included in the meta-analysis of dichotomous effect measures and 11 studies were included in the meta-analysis of continuous effect measures; for acute cellular rejection in adults with exclusion of measurements from the early postoperative period, 8 studies were included in the meta-analysis of dichotomous effect measures and 5 studies were included in the meta-analysis of continuous effect measures.

We identified 1,927 results through the search, and one additional citation through reference lists. After automatic deduplication, we screened 1,499 titles and abstracts. We reviewed full texts of 68 potentially relevant studies, from which 27 were included in this review, with 20 in quantitative form (Figure 1).

### Description of Included Studies

Twenty-seven studies (34–60) involving 1,684 cardiac transplant recipients were included. Detailed characteristics of included studies are reported in Table 1.

**TABLE 1:** Characteristics of included studies.

Study ID	Design	Number of patients, number of samples, and demographic	Age (Mean ± SD, years)	Sex (% male)	Troponin type	Troponin measurement period post transplantation and early measurement exclusions	Troponin measurement method	Troponin threshold (ng/ml) and Selection method	Classification threshold for significant rejection and samples with significant rejection (%)	Outcome(s) and effect measure(s)	Modified QUADAS-2 risk of bias
Ahn (34)	Single Centre Retrospective	47	47.4 ± 15.8	68.1%	TnI, hsTnI Index^a^	2 weeks postoperative onwards	ARCHITECT i2000sr STAT TnI and hsTnI assay (Abbott Diagnostics, Abbott Park, Illinois, USA)	1.17 (hsTnI Index)	ISHLT 2004, 2R	Acute Cellular Rejection	high
		252				Exclusions: none and first 2 months after transplantation		Receiver operating characteristic analysis	7%	Dichotomous and continuous	
		Adult									
Alexis (35)	Single Centre Prospective	90	48.0 ± 15.2	74.4%	TnT	1 week to 72 months postoperative	Enzymun-Test TnT enzyme immunometric assay (Boehringer Mannheim Diagnostics GmbH, Mannheim, Germany)	0.1	ISHLT 1990, 3A	Acute Cellular Rejection	high
		256				Exclusions: none and first 3 months after transplantation		Manufacturer’s recommendation	5%	Dichotomous	
		Adult									
Balduini (36)	Single Centre Prospective	57	37.5 (SD not reported)	Not reported	TnT	1 month to 12 months	Elecsys Electrochemiluminescence Immunoassay (Roche Diagnostics, Indianapolis, Indiana, USA)	Not reported	ISHLT 1990, 1B	Acute Cellular Rejection	unclear
		149				Exclusions: first 1 month after transplantation		Not reported	23%	Continuous	
		Adult									
Cauliez (37)	Single Centre Prospective	56	Not reported	Not reported	TnI	10 to 3,807 days (median 458 days)	Stratus Cardiac TnI fluorometric enzyme immunoassay (Dade-Behring, Newark, Delaware, USA)	0.6	ISHLT 1990, 2	Acute Cellular Rejection	unclear
		100				No exclusions		Manufacturer’s recommendation	9%	Continuous	
		Adult									
Chance (38)	Single Centre Prospective	145	Not reported	Not reported	TnT	3 days to 206 months (median 29 months)	Elecsys Electrochemiluminescence Immunoassay (Roche Diagnostics, Indianapolis, Indiana, USA)	0.1	ISHLT 1990, 3A	Acute Cellular Rejection	unclear
		704				Exclusions: none and first 1 month after transplantation		Manufacturer’s recommendation	20%	Dichotomous and continuous	
		Adult									
Dengler (39)	Single Centre Retrospective	95	48.2 ± 11.4	82.1%	TnT	3 months–70 months (median 15 months)	Enzymun-Test TnT enzyme immunometric assay (Boehringer Mannheim Diagnostics GmbH, Mannheim, Germany)	0.015	ISHLT 1990, 3A	Acute Cellular Rejection	unclear
		271				Exclusions: first 3 months after transplantation		Lower limit of assay detection	17%	Dichotomous and continuous	
		Adult									
Dyer (40)	Single Centre Prospective	42	11.1 (SD not reported)	Not reported	hsTnT	3 months onwards (median 24 months)	Elecsys Electrochemiluminescence Immunoassay (Roche Diagnostics, Indianapolis, Indiana, USA)	0.014	ISHLT 2004, 2R	Acute Cellular Rejection	unclear
		53				Exclusions: first 3 months after transplantation		99th percentile of healthy adult reference population	13%	Dichotomous and continuous	
		Paediatric									
Faulk (41)	Single Centre Prospective	68	30.3 ± 14.2	60.3%	TnT	6 months onwards	Enzymun-Test TnT enzyme immunometric assay (Boehringer Mannheim Diagnostics GmbH, Mannheim, Germany)	0.1	ISHLT 1990, 3A	Acute Cellular Rejection	high
		151				Exclusions: first 6 months after transplantation		Manufacturer’s recommendation	6%	Dichotomous	
		Adult									
Forni (42)	Single Centre Prospective	114	52.0 ± 6.0	86.0%	TnI	15 to 1,740 days (mean 640 ± 95 days)	Dimension Rx L clinical chemistry system (Siemens Medical Solutions Diagnostics, Erlangen, Germany)	0.1	ISHLT 1990, 3A	Acute Cellular Rejection	high
		385				No exclusions		Manufacturer’s recommendation	3%	Dichotomous and continuous	
		Adult									
Garrido (43)	Single Centre Prospective	21	60.0 ± 10.0	81.0%	TnT	1 year onwards	Electrochemiluminescence immunoassays with a Modular Analytics E170 analyzer (Roche Diagnostics GmbH, Mannheim, Germany)	0.026	Not applicable	Cardiac allograft vasculopathy	high
		Not applicable				No exclusions		Receiver operating characteristic analysis		Dichotomous and continuous	
		Adult									
Gleissner (44)	Single Centre Retrospective	132	58.5 ± 9.4	85.6%	TnT	3 months–48 months (mean 13 months)	Enzymun-Test TnT enzyme immunometric assay (Boehringer Mannheim Diagnostics GmbH, Mannheim, Germany)	0.14	ISHLT 1990, 3A	Acute Cellular Rejection	Low
		788				Exclusions: first 3 months after transplantation		Receiver operating characteristic analysis	13%	Dichotomous and continuous	
		Adult									
Halwachs (45)	Single Centre Retrospective	15	49.8 ± 13.6	80.0%	TnT	1 day to 2 years	Enzymun-Test TnT enzyme immunometric assay (Boehringer Mannheim Diagnostics GmbH, Mannheim, Germany)	0.2	ISHLT 1990, 3A	Acute Cellular Rejection	unclear
		183				No exclusions		Manufacturer’s recommendation	1%	Continuous	
		Adult									
Hossein-Nia (48)	Single Centre Prospective	15	Not reported	Not reported	TnT	Postoperative onwards	Enzymun-Test TnT enzyme immunometric assay (Boehringer Mannheim Diagnostics GmbH, Mannheim, Germany)	0.2	ISHLT 1990, 2	Acute Cellular Rejection	low
		65				No exclusions		Manufacturer’s recommendation	16%	Continuous	
		Adult									
Hossein-Nia (46)	Single Centre Prospective	29	48.5 ± 7.8	83.9%	TnT	Postoperative onwards (mean 87 ± 32 weeks)	Enzymun-Test TnT enzyme immunometric assay (Boehringer Mannheim Diagnostics GmbH, Mannheim, Germany)	0.2	ISHLT 1990, 2	Acute Cellular Rejection	unclear
		Not reported				No exclusions		Manufacturer’s recommendation	Not reported	Dichotomous	
		Adult									
Hossein-Nia (47)	Single Centre Prospective	17	Not reported	Not reported	TnI	Postoperative onwards (mean 61 ± 16 days)	TnI Assay (Sanofi Diagnostic Pasteur Ltd., Guildford, United Kingdom)	Not reported	ISHLT 1990, 2	Acute Cellular Rejection	unclear
		214				No exclusions		Not reported	4%	Continuous	
		Adult									
Hsu (49)	Single Centre Prospective	51	47.8 ± 11.3	80.0%	TnI	1 week to 5 years	R&D Systems ELISA (R & D Systems USA, Minneapolis, Minnesota, USA)	Not reported	ISHLT 1990, 2	Acute Cellular Rejection	high
		71				No exclusions		Not reported	23%	Continuous	
		Adult									
Mendez (50)	Multicentre Prospective	73	54.0 ± 14.0	71.2%	hsTnT	Within 3 months of surgery to over 18 months, as needed	Elecsys Electrochemiluminescence Immunoassay (Roche Diagnostics, Indianapolis, Indiana, USA)	0.017	ISHLT 2004, 2R	Acute Cellular Rejection	low
		224				No exclusions		Receiver operating characteristic analysis	7%	Dichotomous and continuous	
		Adult									
Moran (51)	Single Centre Prospective	37	Median 12.4, range 1.3–30.0	Not reported	TnI, TnT	2.05 ± 2.43 years (median, 0.9; range, 0.03–9.1)	Elecsys Electrochemiluminescence Immunoassay (Roche Diagnostics, Indianapolis, Indiana, USA)	TnI: 0.5 Receiver operating characteristic analysis	ISHLT 1990, 3A	Acute Cellular Rejection	high
		85				No exclusions		TnT: Not reported	15%	Dichotomous and continuous	
		Paediatric									
Mullen (52)	Single Centre Prospective	29	52.0 ± 5.4	79.3%	TnI, TnT^b^	12–564 days (mean 129 ± 9 days)	Not reported	Not reported	ISHLT 1990, 3A	Acute Cellular Rejection	low
		173				No exclusions		Not reported	1%	Continuous	
		Adult									
Munoz-Esparza (53)	Single Centre Prospective	72	53.0 ± 13.0	75.0%	hsTnT	Within 1 year	Elecsys Electrochemiluminescence Immunoassay (Roche Diagnostics, Indianapolis, Indiana, USA)	0.035	ISHLT 2004, 2R	Acute Cellular Rejection	high
		Not reported				No exclusions		Receiver operating characteristic analysis	43%	Dichotomous and continuous	
		Adult									
Ogawa (54)	Multicentre Prospective	69	50.0 ± 10.0	79.7%	TnT	9–141 weeks (mean 53 ± 26 weeks)	Elecsys Electrochemiluminescence Immunoassay (Roche Diagnostics, Indianapolis, Indiana, USA)	Not reported	ISHLT 1990, 3A	Acute Cellular Rejection	unclear
		683				No exclusions		Not reported	4%	Continuous	
		Adult									
Patel (55)	Multicentre Retrospective	98	53.8 ± 12.1	83.0%	hsTnI	1 week—long term (median 1522 (IQR 773–2160) days)	ARCHITECT i2000sr STAT high-sensitivity cTnI assay (Abbott Diagnostics, Abbott Park, Illinois, USA)	0.015	ISHLT 2004, 2R	Acute Cellular Rejection	unclear
		418				No exclusions		Receiver operating characteristic analysis	5%	Dichotomous and continuous	
		Adult									
Siaplaouras (56)	Single Centre Retrospective	25	Mean 2 months, range 2 weeks–13 years	40.0%	TnI	3 weeks to 4 years	Stratus Cardiac TnI fluorometric enzyme immunoassay (Dade-Behring, Newark, Delaware, USA)	0.6	ISHLT 1990, 3A	Acute Cellular Rejection	high
		Not reported				No exclusions		Manufacturer’s recommendation	Not reported	Dichotomous	
		Paediatric									
Vazquez-Rodriguez (57)	Single Centre Prospective	62	Not reported	85.5%	TnT	Postoperative onwards	Enzymun-Test TnT enzyme immunometric assay (Boehringer Mannheim Diagnostics GmbH, Mannheim, Germany)	0.1	ISHLT 1990, 2	Acute Cellular Rejection	low
		259				Exclusions: None and first 3 months after transplantation		Manufacturer’s recommendation	25%	Dichotomous	
		Adult									
Wåhlander (58)	Single Centre Prospective	14	Not reported	Not reported	TnI	1 month onwards	Elecsys Electrochemiluminescence Immunoassay (Roche Diagnostics, Indianapolis, Indiana, USA)	0.1	ISHLT 1990, 3A	Acute Cellular Rejection	unclear
		78				Exclusions: first 1 month after transplantation		Manufacturer’s recommendation	12%	Dichotomous and continuous	
		Paediatric									
Walpoth (59)	Single Centre Prospective	25	Not reported	Not reported	TnT	Postoperative to 2 years	Enzymun-Test TnT enzyme immunometric assay (Boehringer Mannheim Diagnostics GmbH, Mannheim, Germany)	0.2	Texas score, 4	Acute Cellular Rejection	unclear
		392				No exclusions		Manufacturer’s recommendation	Not reported	Continuous	
		Adult									
Wang (60)	Single Centre Prospective	186	Not reported	Not reported	TnI, TnT^b^	Postoperative onwards	TnI: Stratus Cardiac TnI fluorometric enzyme immunoassay (Dade-Behring, Newark, Delaware, USA)	TnI: 1.7 Not reported	ISHLT 1990, 3A	Acute Cellular Rejection	high
		358				Exclusions: first 5 weeks after transplantation	TnT: Enzymun-Test TnT enzyme immunometric assay (Boehringer Mannheim Diagnostics GmbH, Mannheim, Germany)	TnT: 0.07 Not reported	21%	Dichotomous and continuous	
		Adult									

a Where studies measured both conventional and high sensitivity troponin variants and underwent meta-analysis, high sensitivity troponin was included in quantitative analysis where appropriate.

b Where studies measured both troponin I and T subtypes and underwent meta-analysis, troponin I measurements was chosen for quantitative synthesis and a sensitivity analysis was performed by including troponin T measurements to determine the impact of this decision. TnT, Troponin T; TnI, Troponin I; hsTnT, High Sensitivity Troponin T; hsTnI, High Sensitivity Troponin I.

### Methodological Quality

Methodological quality was variable. Five studies (44, 48, 50, 52, 57) were deemed low risk of bias, 12 studies (36–40, 45–47, 54, 55, 58, 59) unclear risk of bias due to no specific reporting of certain domain characteristics, and 10 studies (34, 35, 41–43, 49, 51, 53, 56, 60) high risk of bias. The full QUADAS-2 assessment can be found in the Supplementary Material.

### Descriptive Analyses and Meta-Analysis

#### Acute Cellular Rejection

##### Adult

###### No Temporal Exclusion Criteria

####### Dichotomous Measure of Diagnostic Accuracy

**FIGURE 2:**
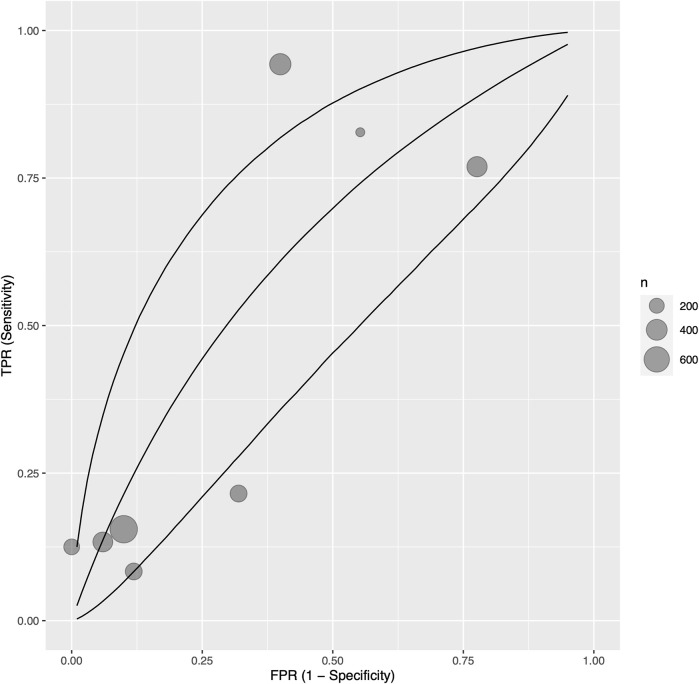
Bayesian summary receiver operating characteristic curve showing summary diagnostic accuracy of recipient troponin in acute rejection with no temporal exclusions, with upper and lower 95% credible bands. Each filled circle represents one included study, the size of which is weighted in proportion to the study’s sample size.

Eight studies (35, 38, 42, 50, 53, 55, 57, 60) with 840 participants reported sensitivity, specificity, and AUC values regarding the ability of troponin to diagnose acute cellular rejection in heart transplant recipients. We found a pooled sensitivity of 0.479 (95% CrI 0.190–0.783), specificity of 0.702 (95% CrI 0.395–0.910), and BAUC 0.584 (95% CrI 0.377–0.760) (Figure 2).

As one included study (60) measured both troponin I and T values, we performed a sensitivity analysis investigating the effects of including troponin T measurements instead of troponin I in quantitative synthesis. The result was not significantly different; pooled sensitivity was 0.498 (95% CrI 0.206–0.788), specificity 0.696 (95% CrI 0.387–0.901), and BAUC 0.591 (95% CrI 0.385–0.758) (Supplementary Figure S1).

Hossein-Nia 1995 (46) reported sensitivity of 0.333 but did not report a corresponding specificity.

We investigated potential sources of statistical heterogeneity with a meta-regression, and found that the troponin assay sensitivity and ISHLT rejection criteria, study year, and number of study centres were significant AUC modifiers (Supplementary Table S1). In particular, studies which used high sensitivity troponin assays were also those which used the ISHLT 2004 criteria, and this was associated with a 0.210 increased AUC (*p* = 0.0006) (Supplementary Figure S2). A unit increase in study year was associated with an increased AUC of 0.014 (*p* = 0.0010), and a multicentre study design was associated with an increased AUC of 0.189 (*p* = 0.0154) compared to a single centre design (Supplementary Figure S3). Notably, the following were not significant AUC modifiers: ISHLT cut-off grade for definition of significant rejection (1R vs. 2R in ISHLT 2004; 2 vs. 3A in ISHLT 1990), prevalence of samples with significant rejection per cohort, troponin threshold, and study risk of bias.

####### Continuous Measure of Diagnostic Accuracy

**FIGURE 3:**
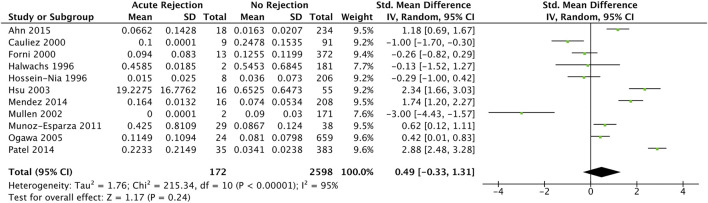
Forest plot of standardised mean differences for elevated recipient troponin in diagnosing acute rejection post cardiac transplantation, with no temporal exclusions.

Eleven studies (34, 37, 42, 45, 47, 49, 50, 52–55) with 641 participants reported troponin mean differences between those with and without acute cellular rejection. We found that the standardised troponin measurements were not significantly different in those with and without acute cellular rejection (SMD 0.49, 95% CI −0.33–1.31) (Figure 3).

As one included study (52) measured both troponin I and T values, we performed a sensitivity analysis investigating the effects of including troponin T measurements instead of troponin I in quantitative synthesis. The result was not significantly different (pooled SMD 0.26, 95% CI −0.64–1.16) (Supplementary Figure S4).

Wang 1996 (60) reported mean measurements in both troponin I and T between acute cellular rejection vs. non-rejection groups (0.216 vs. 0.707 and 0.134 vs. 0.088 ng/ml respectively); however, neither were statistically significant (*p* = 0.357 and *p* = 0.374 respectively). Contrary to this, Walpoth 1998 (59) reported statistically significant elevations (no measure of statistical significance reported) troponin T measurements between acute cellular rejection (0.77 ± 0.80 ng/ml) and non-rejection (0.02 ± 0.05 ng/ml) groups. Hossein-Nia 1993 (48) reported an elevated median troponin T in those with acute cellular rejection compared to without (0.370 vs. 0.300 ng/ml); however, statistical significance was not reported.

Between-study statistical heterogeneity was considerable (I^2^ statistic 95%). We investigated potential sources of statistical heterogeneity with a meta-regression, and found that the troponin assay sensitivity and ISHLT rejection criteria (overlapping exactly; all studies using high sensitivity troponin also used ISHLT 2004 criteria), study year, troponin threshold, and standard deviation of age were significant SMD modifiers and accounted for up to 49% of heterogeneity on univariable analysis (Supplementary Table S2). Notably, the following were not significant SMD modifiers: ISHLT cut-off grade for definition of significant rejection (1R vs. 2R in ISHLT 2004; 2 vs. 3A in ISHLT 1990), prevalence of samples with significant rejection per cohort, and study risk of bias.

A regression test for funnel plot asymmetry was unable to detect significant publication bias (*p* = 0.1023) (Supplementary Figure S5).

###### Early Postoperative Exclusion Criteria

####### Dichotomous Measure of Diagnostic Accuracy

**FIGURE 4:**
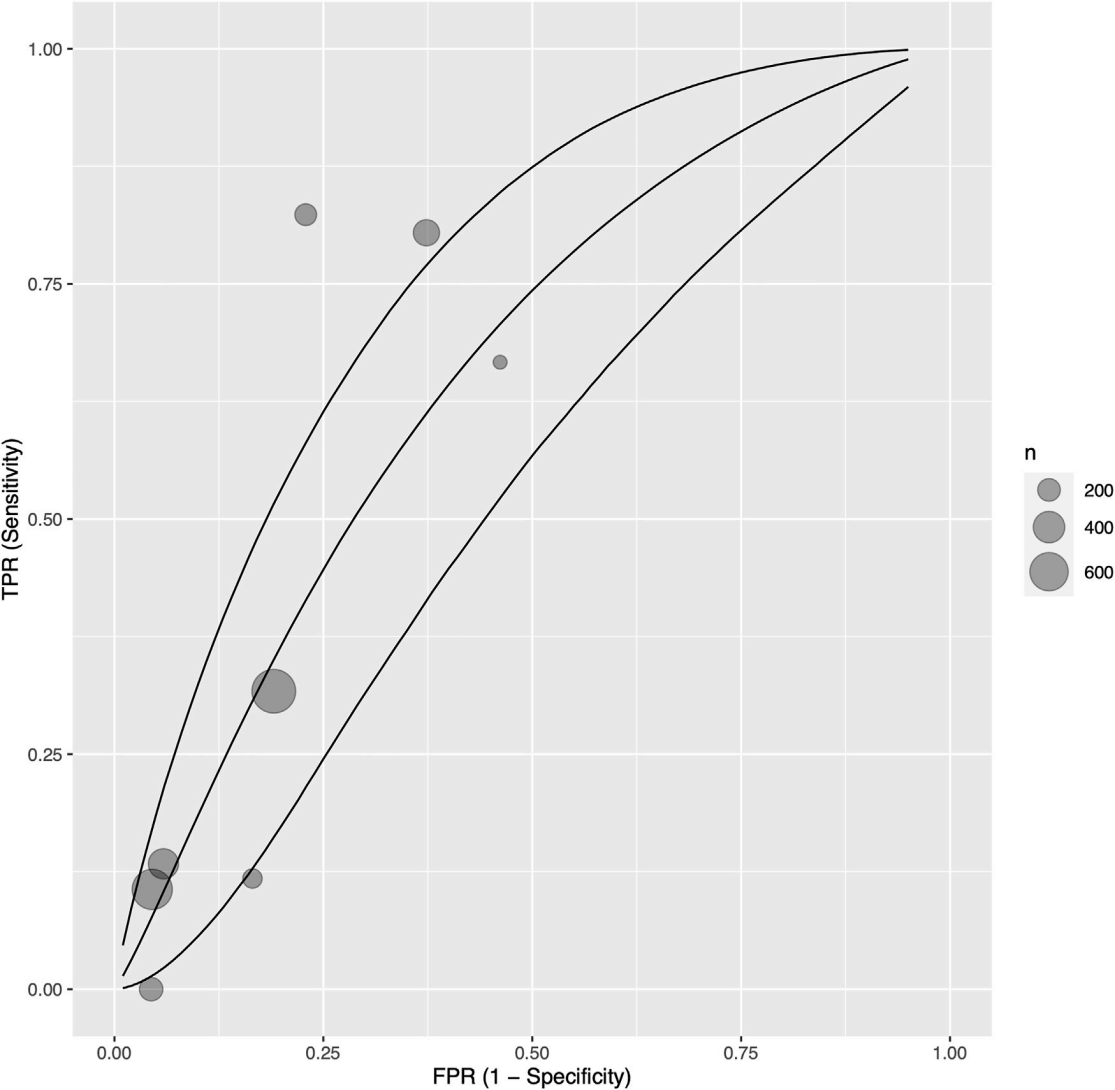
Bayesian summary receiver operating characteristic curve showing summary diagnostic accuracy of recipient troponin in acute rejection with early postoperative measurements (at least 1 month postoperative) excluded, with upper and lower 95% credible bands. Each filled circle represents one included study, the size of which is weighted in proportion to the study’s sample size.

After exclusion of measurements from the early postoperative period (at least 1 month postoperatively), eight single centre studies (34, 35, 38, 39, 41, 44, 57, 60) with 825 participants reported sensitivity, specificity, and AUC values regarding the ability of troponin to diagnose acute cellular rejection in heart transplant recipients. We found a pooled sensitivity of 0.414 (95% CrI 0.174–0.696), specificity of 0.785 (95% CrI 0.567–0.912), and BAUC 0.607 (95% CrI 0.469–0.723) (Figure 4).

We investigated potential sources of statistical heterogeneity with a meta-regression, and found that the troponin assay sensitivity and ISHLT rejection criteria, and troponin type, and study design were significant AUC modifiers (Supplementary Table S3). In particular, use of high sensitivity troponin I assays by one study (34) corresponded exactly to use of ISHLT 2004 criteria, and was associated with a 0.257 increase in AUC (*p* = 0.0270) (Supplementary Figure S6). Of note, the length of early postoperative exclusion (from 1 month to 6 months) was not associated with significant changes to troponin’s diagnostic ability. Additionally, the following were not significant SMD modifiers: ISHLT cut-off grade for definition of significant rejection (1R vs. 2R in ISHLT 2004; 2 vs. 3A in ISHLT 1990), prevalence of samples with significant rejection per cohort, troponin threshold, and study risk of bias.

####### Continuous Measure of Diagnostic Accuracy

**FIGURE 5:**
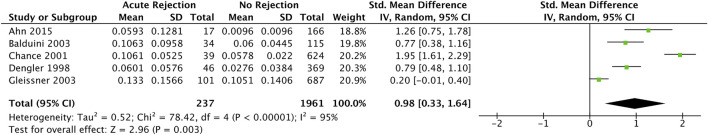
Forest plot of standardised mean differences for elevated recipient troponin in diagnosing acute rejection post cardiac transplantation, with early postoperative measurements (at least 1 month postoperative) excluded.

Five studies (34, 36, 38, 39, 44) with 476 participants reported troponin mean differences between those with and without acute cellular rejection. We found that the standardised troponin measurements were higher in those with acute cellular rejection, and that this was a large and statistically significant effect (SMD 0.98, 95% CI 0.33–1.64) (Figure 5).

Wang 1996 (60) reported mean measurements in both troponin I and T between acute cellular rejection vs. non-rejection groups (0.059 vs. 0.102 and 0.069 vs. 0.044 ng/ml respectively) after measurements during the first 5 weeks were excluded; however, neither were statistically significant (*p* = 0.713 and *p* = 0.382 respectively).

Statistical heterogeneity was considerable (I^2^ statistic 95%); however, meta-regression was not possible due to insufficient study numbers (*n* = 5).

##### Paediatric

###### No Temporal Exclusion Criteria

Two studies (51, 56) with 62 participants investigated the association between troponin and adverse outcomes in cardiac transplantation recipients. Moran 2000 (51) found that troponin I values differed significantly across ISHLT 1990 grades 0, 1, 2, and 3 on analysis of variance (ANOVA) (*p* = 0.034), with a diagnostic sensitivity of 0.500 and specificity of 0.776. However, troponin T values were not significantly different across ISHLT 1990 grades 0, 1, 2, and 3 on ANOVA (*p* = 0.16)—sensitivity was 0.421 and specificity was 0.837. Siaplaouras 2003 (56) found a sensitivity of 0.750, but did not report a corresponding specificity.

###### Early Postoperative Exclusion Criteria

After exclusion of measurements from the early postoperative period (at least 1 month postoperatively), three studies (40, 56, 58) with 81 participants studied the association between troponin and adverse outcomes in cardiac transplantation recipients. Excluding measurements from the first 3 months after transplantation, Dyer 2012 (40) reported a statistically significant elevation in high sensitivity troponin T values in those with acute cellular rejection (SMD 2.44, 95% CI 1.51–3.37), and a sensitivity of 0.857 and specificity of 0.913. Wåhlander 2002 (58) found that conventional troponin T values were also elevated in those with acute cellular rejection (SMD 1.43, 95% CI 0.70–2.17), reporting a sensitivity of 0.556 and specificity of 0.768. Siaplaouras 2003 (56) found a sensitivity of 0.591, but did not report a corresponding specificity.

## Discussion

In this systematic review and meta-analysis of 27 diagnostic observational studies involving over 1,600 patients, we provide the most up-to-date evidence of the value of troponin in diagnosing adverse outcomes in heart transplant recipients. We found that late troponin levels (measured at least 1 month postoperatively) were significantly elevated in adult recipients with acute cellular rejection. Diagnostic accuracy of plasma troponin was slightly higher for measurements taken after the early postoperative period compared to those taken across all postoperative periods; however, the diagnostic ability of both were poor.

The poor diagnostic utility of troponin in the immediate to early post-operative period in detecting acute cellular rejection is not surprising given the manifold pathologies that can drive elevated plasma troponin in this setting (61). Our results suggest that this “early” post-operative period is confined to 1 month, with no significant difference in diagnostic accuracy of troponins measured after 1 month compared to 6 months post-transplant. However, risk of rejection is also highest in the first months after transplant (62), coinciding with this period of poorer diagnostic utility. Biomarkers capable of distinguishing between early acute rejection and routine perioperative cardiac injury are needed.

Additionally, our meta-regressions suggest that the utility of troponin may be improving over time, with study year being positive effect modifier in multiple analyses. While this is possibly attributable to the transition to high-sensitivity troponin assays, these findings are also confounded by a perfect overlap with a transition to the ISHLT 2004 criteria for acute cellular rejection.

Our search revealed one other systematic review, without meta-analysis, on a similar topic (63). However, this literature search excluded key databases (Embase and the Cochrane Library) and therefore may have lacked sensitivity, with only 88 abstracts identified and 12 studies included in the final analysis; there was no formal assessment of methodological quality; and there was no quantitative meta-analysis or assessment and management of potential sources of heterogeneity. Hence, we believe our study adds to the existing knowledge base, and provides the most recent and high-quality synthesis regarding the diagnostic value of cardiac troponin in heart transplant recipients.

Our review should be interpreted with the following limitations. While five studies were identified to be at low risk of bias, the 22 remaining studies were at unclear or high risk of bias; reassuringly though, study risk of bias was not found to be a significant effect modifier in all meta-regressions where this was possible. Studies did not report timing of troponin sample procurement—before vs. after EMB—despite this being a possible confounder as procedure related injury can occur. The majority of studies were single centre, raising potential concerns for external validity. Finally, despite our efforts in determining significant sources of statistical heterogeneity, we were not able to account for all observed statistical heterogeneity. Although our prespecified use of a random-effects model is a strength of our study design, our findings are nonetheless tempered by unaccounted heterogeneity—an inherent part of meta-analysis methodology—which may be attributable to systematic differences in unreported study baseline characteristics as well as other study and patient-level factors. Large, high quality, randomised studies would be needed to control for these unmeasured factors in particular.

In accordance with international guidelines (21, 22), our results do not support the routine use of troponin for surveillance or diagnosis of acute cellular rejection. However, our work identifies many opportunities for future research. The current gold standard diagnostic test for acute cellular rejection involves an invasive EMB which exposes patients to small but significant risks (3–5) and can be associated with poor pathological concordance (2); safer and more effective diagnostic tests are urgently needed. While numerous imaging parameters and biomarkers have been investigated for this purpose, donor-derived cell-free DNA has recently emerged as a promising, non-invasive marker of acute rejection detection (64). Not only is donor-derived cell-free DNA able to detect episodes of rejection with remarkable sensitivity and specificity, but it may also be able to distinguish between acute cellular rejection and antibody mediated rejection, at time points earlier than possible with EMBs (65). As accurate as any one diagnostic marker may be however, experience from multiple disciplines has supported the implementation of well-selected multi-biomarker diagnostic panels over any singular biomarker (66–68). Accordingly, opportunity exists to assess elevated high-sensitivity cardiac troponin—a sensitive and specific marker of the cardiomyocyte death which occurs during acute rejection—in conjunction with emerging biomarkers representing different pathophysiological aspects of acute cellular rejection to optimise the “liquid biopsy” approach and reduce uncertainty and clinical risk of the current EMB approach. While the diagnostic ability of troponin (in the early postoperative month in particular) as a single parameter is insufficient to warrant implementation, whether or not its diagnostic utility can be enriched by integration into sophisticated multivariable diagnostic models with other non-invasive haematological and clinical markers is a field with significant potential. High-sensitivity troponin in particular may possess sufficiently high negative predictive value aid in ruling out acute cellular rejection (55, 63). Additionally, in order to optimise methodological quality and minimise risk of study bias, future researchers should design and report diagnostic test accuracy studies in accordance with QUADAS-2, among other design and reporting guidelines.

## Conclusion

In this systematic review and meta-analysis, we found a positive association between late troponin elevation and acute cellular rejection in adults, however diagnostic performance was insufficient to support its routine use in the diagnostic pathway. Further research is warranted to assess whether the addition of troponin to emerging biomarkers of acute cellular rejection, such as circulating cell-free DNA, could lead to an enhanced “liquid biopsy” capable of superseding invasive testing.

## Abbreviations

ANOVA, analysis of variance; AUC, area under the ROC curve; BAUC, Bayesian AUC; BSROC, Bayesian summary ROC; CI, confidence interval; CrI, credible interval; EMB, endomyocardial biopsy; ISHLT, International Society for Heart and Lung Transplantation; MOOSE, meta-analysis of observational studies in epidemiology; QUADAS-2, quality assessment of diagnostic accuracy studies 2; ROC, receiver operating characteristic; SMD, standardised mean difference.
