# Supplementary material for: Elevated Cardiac Troponin to Detect Acute Cellular Rejection After Cardiac Transplantation: A Systematic Review and Meta-Analysis
Source: Transpl Int. 2022 Jun 8;35:10362. doi: 10.3389/ti.2022.10362 (PMC9215116; doi:10.3389/ti.2022.10362)
Supplement: Supplementary file 1 [file DataSheet1.PDF]

Elevated Cardiac Troponin to Detect Acute Cellular Rejection after Cardiac Transplantation: A Systematic Review and Meta-Analysis

Online Supplement

Table of Contents

Search Strategy *2*

MEDLINE 2

Embase 2

Cochrane Library 2

Statistical Analysis and Data Synthesis *3*

Bivariate Bayesian modelling details *4*

Prior distributions 4

Other modelling details 4

Prior-to-posterior sensitivity analyses 4

Prior-to-posterior sensitivity analysis for Acute Cellular Rejection – Adult – No Temporal Exclusion Criteria – Dichotomous Measure of Diagnostic Accuracy 5

Prior-to-posterior sensitivity analysis for Acute Cellular Rejection – Adult – Early Postoperative Exclusion Criteria – Dichotomous Measure of Diagnostic Accuracy 7

Meta-regression Covariates *9*

Assessment of Methodological Quality *10*

Meta-Regression eTables *11*

eTable 1 – Acute Rejection, Adult, No Temporal Exclusion Criteria, Dichotomous 11

eTable 2 – Acute Rejection, Adult, No Temporal Exclusion Criteria, Continuous 12

eTable 3 – Acute Rejection, Adult, Early Postoperative Exclusion Criteria, Dichotomous 13

eFigures *14*

eFigure 1 14

eFigure 2 15

eFigure 3 16

eFigure 4 17

eFigure 5 18

eFigure 6 19

Supplement References *20*

Search Strategy

MEDLINE

1. exp Troponin/ or troponin.mp.
2. exp Heart Transplantation/
3. ((heart* adj3 transplant*) or (cardiac adj3 transplant*)).mp.
4. 2 or 3
5. 1 and 4

Embase

1. exp troponin/ or troponin.mp.
2. exp heart transplantation/
3. ((heart* adj3 transplant*) or (cardiac adj3 transplant*)).mp.
4. 2 or 3
5. 1 and 4

Cochrane Library

#1 troponin
#2 MeSH descriptor: [Troponin] explode all trees
#3 MeSH descriptor: [Heart Transplantation] explode all trees
#4 (heart* NEAR/3 transplant*) OR (cardiac NEAR/3 transplant*)
#5 #1 OR #2
#6 #3 OR #4
#7 #5 AND #6

Statistical Analysis and Data Synthesis

Some studies [1-11] excluded measurements taken early following cardiac transplantation (ranging from the first 1 to 6 months, as defined by individual studies) to investigate the association between late troponin elevation (measured at least 1 month postoperative) and acute cellular rejection. This is due to reports of elevated troponin levels in both acute cellular rejection and non-rejection patients during the early postoperative period [12]. We conducted separate analyses for studies with and without this early postoperative exclusion criteria of at least 1 month. We also conducted separate analyses for adult and paediatric populations. For outcomes where meta-analysis could not be performed due to insufficient study number, variable study-level reporting, or significant inter-study heterogeneity, we explored reported data without the use of formal statistical meta-analysis methods.

We report prior distributions, chains, iterations, burn-ins, and other modelling details, and perform prior-to-posterior sensitivity analyses for our results in the following pages. Prior-to-posterior sensitivity analyses did not significantly affect our results; all point estimates were similar and 95% credible intervals were overlapping.

Where studies measured both conventional and high sensitivity troponin variants, high sensitivity troponin was included in quantitative analysis. Where studies measured both troponin I and T subtypes, troponin I measurements were chosen for quantitative synthesis and a sensitivity analysis was performed by including troponin T measurements to determine the impact of this decision.

Bivariate Bayesian modelling details

Prior distributions

For the bivariate Bayesian modelling we implemented, priors were specified for the hyperparameters μ_D, μ_S, σ_D, σ_S, ρ, and U [13]:

- $\mu_D \sim$ Logistic(0,1)
- $\mu_S \sim$ Logistic(0,1)
- $\sigma_D \sim$ Uniform(0,10)
- $\sigma_S \sim$ Uniform(0,10)
- ρ in the Fisher scale ~ Normal$(0,\frac{1}{\sqrt{2}})$
- U ~ Uniform$(\frac{1}{30},\frac{1}{3})$

Other modelling details

- Number of chains for Markov chain Monte Carlo computations: 5
- Number of iterations after adapting the Markov chain Monte Carlo: 10,000
- Number of iterations in the adapting process: 1,000
- Number of iterations discarded for burn-in period: 1,000
- Thinning rate: 1

Prior-to-posterior sensitivity analyses

We performed prior-to-posterior sensitivity analyses of the Fisher transformed ρ by giving different values of mean and standard deviation to understand their influence on the analysis. This is because according to the creator of this modelling [13], ρ is the most difficult parameter to estimate within this model, and is also the one parameter he recommends such a sensitivity analysis on.

Prior-to-posterior sensitivity analysis for Acute Cellular Rejection – Adult – No Temporal Exclusion Criteria – Dichotomous Measure of Diagnostic Accuracy

ρ in the Fisher scale	Mean	Standard deviation	BAUC	BAUC 95% CrI lower	BAUC 95% CrI upper	Sensitivity	Sensitivity 95% CrI lower	Sensitivity 95% CrI upper	Specificity	Specificity 95% CrI lower	Specificity 95% CrI upper
Main Analysis	*0*	*1*	*0.584*	*0.377*	*0.760*	*0.479*	*0.190*	*0.783*	*0.702*	*0.395*	*0.910*
Sensitivity Analysis 1	-10	0.1	0.498	0.417	0.561	0.461	0.254	0.745	0.597	0.222	0.843
Sensitivity Analysis 2	-10	0.5	0.511	0.439	0.563	0.454	0.237	0.649	0.625	0.409	0.840
Sensitivity Analysis 3	-10	1	0.573	0.530	0.590	0.349	0.178	0.575	0.791	0.568	0.908
Sensitivity Analysis 4	-10	3	0.557	0.338	0.740	0.480	0.215	0.757	0.659	0.279	0.912
Sensitivity Analysis 5	-10	5	0.571	0.352	0.752	0.480	0.199	0.771	0.682	0.332	0.911
Sensitivity Analysis 6	-5	0.1	0.488	0.272	0.705	0.462	0.282	0.664	0.565	0.165	0.921
Sensitivity Analysis 7	-5	0.5	0.502	0.276	0.716	0.474	0.262	0.701	0.575	0.173	0.921
Sensitivity Analysis 8	-5	1	0.525	0.300	0.723	0.479	0.240	0.729	0.609	0.213	0.917
Sensitivity Analysis 9	-5	3	0.567	0.338	0.748	0.478	0.200	0.767	0.676	0.315	0.911
Sensitivity Analysis 10	-5	5	0.572	0.360	0.748	0.477	0.192	0.767	0.687	0.343	0.910

Sensitivity Analysis 11	0	0.1	0.586	0.383	0.759	0.479	0.191	0.785	0.705	0.397	0.908
Sensitivity Analysis 12	0	0.5	0.584	0.374	0.757	0.477	0.184	0.782	0.703	0.401	0.908
Sensitivity Analysis 13	0	3	0.578	0.362	0.758	0.482	0.191	0.786	0.692	0.366	0.909
Sensitivity Analysis 14	0	5	0.578	0.360	0.753	0.480	0.189	0.781	0.691	0.352	0.909
Sensitivity Analysis 15	5	0.1	0.611	0.482	0.735	0.532	0.156	0.873	0.697	0.455	0.882
Sensitivity Analysis 16	5	0.5	0.613	0.445	0.756	0.525	0.136	0.868	0.714	0.479	0.888
Sensitivity Analysis 17	5	1	0.608	0.419	0.769	0.507	0.138	0.854	0.723	0.478	0.895
Sensitivity Analysis 18	5	3	0.589	0.388	0.760	0.483	0.176	0.798	0.708	0.401	0.907
Sensitivity Analysis 19	5	5	0.581	0.369	0.753	0.484	0.190	0.790	0.694	0.362	0.906
Sensitivity Analysis 20	10	0.1	0.606	0.542	0.649	0.537	0.276	0.753	0.686	0.506	0.828
Sensitivity Analysis 21	10	0.5	0.589	0.529	0.687	0.505	0.282	0.797	0.693	0.517	0.817
Sensitivity Analysis 22	10	1	0.576	0.521	0.671	0.456	0.198	0.849	0.710	0.413	0.879
Sensitivity Analysis 23	10	3	0.596	0.397	0.763	0.492	0.169	0.816	0.713	0.436	0.901

BAUC = Bayesian area under the receiver operating characteristic curve, CrI = Credible interval

Prior-to-posterior sensitivity analysis for Acute Cellular Rejection – Adult – Early Postoperative Exclusion Criteria – Dichotomous Measure of Diagnostic Accuracy

**ρ in the Fisher scale**	**Mean**	**Standard deviation**	**BAUC**	**BAUC 95% CrI lower**	**BAUC 95% CrI upper**	**Sensitivity**	**Sensitivity 95% CrI lower**	**Sensitivity 95% CrI upper**	**Specificity**	**Specificity 95% CrI lower**	**Specificity 95% CrI upper**
Main Analysis	*0*	*1*	*0.607*	*0.469*	*0.723*	*0.414*	*0.174*	*0.696*	*0.785*	*0.567*	*0.912*
Sensitivity Analysis 1	-10	0.1	0.528	0.476	0.581	0.628	0.277	0.819	0.470	0.223	0.840
Sensitivity Analysis 2	-10	0.5	0.541	0.394	0.593	0.511	0.373	0.602	0.617	0.349	0.799
Sensitivity Analysis 3	-10	1	0.565	0.507	0.591	0.377	0.218	0.634	0.761	0.444	0.881
Sensitivity Analysis 4	-10	3	0.596	0.473	0.698	0.406	0.207	0.659	0.775	0.559	0.901
Sensitivity Analysis 5	-10	5	0.615	0.474	0.729	0.421	0.159	0.730	0.793	0.597	0.908
Sensitivity Analysis 6	-5	0.1	0.607	0.559	0.637	0.403	0.184	0.686	0.785	0.532	0.924
Sensitivity Analysis 7	-5	0.5	0.608	0.566	0.643	0.399	0.209	0.634	0.791	0.593	0.911
Sensitivity Analysis 8	-5	1	0.610	0.512	0.645	0.409	0.205	0.664	0.785	0.526	0.918
Sensitivity Analysis 9	-5	3	0.612	0.468	0.733	0.424	0.165	0.737	0.787	0.564	0.908
Sensitivity Analysis 10	-5	5	0.620	0.473	0.739	0.432	0.158	0.758	0.792	0.598	0.908
Sensitivity Analysis 11	0	0.1	0.595	0.440	0.710	0.408	0.183	0.683	0.773	0.529	0.911
Sensitivity Analysis 12	0	0.5	0.600	0.453	0.718	0.408	0.177	0.695	0.779	0.546	0.914

Sensitivity Analysis 13	0	3	0.621	0.482	0.732	0.431	0.158	0.743	0.794	0.606	0.906
Sensitivity Analysis 14	0	5	0.623	0.481	0.741	0.438	0.156	0.762	0.794	0.610	0.908
Sensitivity Analysis 15	5	0.1	0.645	0.519	0.758	0.477	0.143	0.830	0.793	0.611	0.911
Sensitivity Analysis 16	5	0.5	0.646	0.514	0.768	0.483	0.146	0.837	0.789	0.620	0.908
Sensitivity Analysis 17	5	1	0.642	0.516	0.758	0.469	0.152	0.819	0.795	0.627	0.908
Sensitivity Analysis 18	5	3	0.634	0.503	0.753	0.456	0.161	0.808	0.793	0.608	0.904
Sensitivity Analysis 19	5	5	0.624	0.486	0.743	0.434	0.154	0.773	0.798	0.620	0.907
Sensitivity Analysis 20	10	0.1	0.644	0.583	0.690	0.506	0.293	0.702	0.769	0.664	0.859
Sensitivity Analysis 21	10	0.5	0.623	0.572	0.650	0.398	0.213	0.631	0.815	0.665	0.894
Sensitivity Analysis 22	10	1	0.642	0.604	0.705	0.494	0.320	0.673	0.775	0.680	0.854
Sensitivity Analysis 23	10	3	0.628	0.539	0.714	0.428	0.172	0.725	0.804	0.667	0.903
Sensitivity Analysis 24	10	5	0.63	0.525	0.732	0.455	0.181	0.765	0.786	0.617	0.901

BAUC = Bayesian area under the receiver operating characteristic curve, CrI = Credible interval

Meta-regression Covariates

Where reporting of pre-specified covariates was sufficient across included studies, we used meta-regressions to explore possible sources of heterogeneity through inputting these covariates as modifier variables into the random effects model. We pre-specified the following covariates for meta-regression: study characteristics such as prospective vs retrospective design, single vs multicentre, methodological quality as per QUADAS-2, publication year, number of participants and samples, male prevalence, and proportion of samples with significant rejection; patient attributes such as age, and BMI; clinical features such as indication for transplantation, International Society for Heart and Lung Transplantation (ISHLT) system of acute cellular rejection grading, threshold for acute rejection classification, and immunosuppressive regimen; perioperative and operative characteristics such as baseline renal function and total ischaemia time; troponin measurement factors such as troponin type, sensitivity of troponin assay, and threshold for elevated troponin; and patient comorbidities such as prevalence of smoking, hypertension, diabetes mellitus, and chronic kidney disease.

Assessment of Methodological Quality

QUADAS-2	OVERALL ASSESSMENT	Patient Selection				Index Test			Reference Standard			Flow and Timing				
**Study**		Was a consecutive or random sample of patients enrolled?	Was a case-control design avoided?	Did the study avoid inappropriate exclusions?	TOTAL	Were the index test results interpreted without knowledge of the results of the reference standard?	If a threshold was used, was it pre-specified?	TOTAL	Is the reference standard likely to correctly classify the target condition?	Were the reference standard results interpreted without knowledge of the results of the index test?	TOTAL	Was there an appropriate interval between index test(s) and reference standard?	Did all patients receive a reference standard?	Did patients receive the same reference standard?	Were all patients included in the analysis?	TOTAL
Ahn 2015	high	Y	Y	Y	low	Y	N	high	Y	Y	low	Y	Y	Y	N	high
Alexis 1998	high	Y	Y	Y	low	Y	Y	low	Y	Y	low	Y	Y	Y	N	high
Balduini 2003	unclear	unclear	Y	Y	unclear	Y	Y	low	Y	Y	low	Y	Y	Y	Y	low
Cauliez 2000	unclear	unclear	Y	Y	unclear	Y	Y	low	Y	Y	low	Y	Y	Y	Y	low
Chance 2001	unclear	unclear	Y	Y	unclear	Y	Y	low	Y	Y	low	Y	Y	Y	Y	low
Dengler 1998	unclear	unclear	Y	Y	unclear	Y	Y	low	Y	Y	low	Y	Y	Y	Y	low
Dyer 2012	unclear	unclear	Y	Y	unclear	Y	Y	low	Y	Y	low	Y	Y	Y	Y	low
Faulk 1998	high	N	Y	Y	high	Y	Y	low	Y	Y	low	Y	Y	Y	Y	low
Forni 2000	high	N	Y	Y	high	Y	Y	low	Y	Y	low	Y	Y	Y	Y	low
Garrido 2012	high	unclear	Y	Y	unclear	Y	N	high	Y	Y	low	Y	Y	Y	Y	low
Gleissner 2003	low	Y	Y	Y	low	Y	Y	low	Y	Y	low	Y	Y	Y	Y	low
Halwachs 1996	unclear	unclear	Y	Y	unclear	Y	Y	low	Y	Y	low	unclear	Y	Y	Y	unclear
Hossein-Nia 1993	low	Y	Y	Y	low	Y	Y	low	Y	Y	low	Y	Y	Y	Y	low
Hossein-Nia 1995	unclear	unclear	Y	Y	unclear	Y	Y	low	Y	Y	low	unclear	Y	Y	Y	unclear
Hossein-Nia 1996	unclear	unclear	Y	Y	unclear	Y	Y	low	Y	Y	low	unclear	Y	Y	Y	unclear
Hsu 2003	high	unclear	Y	Y	unclear	Y	N	high	Y	Y	low	Y	Y	Y	N	high
Mendez 2014	low	Y	Y	Y	low	Y	Y	low	Y	Y	low	Y	Y	Y	Y	low
Moran 2000	high	N	N	Y	high	Y	N	high	Y	Y	low	Y	Y	Y	Y	low
Mullen 2002	low	Y	Y	Y	low	Y	Y	low	Y	Y	low	Y	Y	Y	Y	low
Munoz-Esparza 2011	high	N	Y	Y	high	Y	Y	low	Y	Y	low	Y	Y	Y	unclear	unclear
Ogawa 2005	unclear	unclear	Y	Y	unclear	Y	Y	low	Y	Y	low	Y	Y	Y	Y	low
Patel 2014	unclear	unclear	Y	Y	unclear	Y	Y	low	Y	Y	low	Y	Y	Y	Y	low
Siaplaouras 2003	high	Y	Y	Y	low	Y	unclear	unclear	Y	Y	low	Y	Y	Y	N	high
Vazquez-Rodriguez 1999	low	Y	Y	Y	low	Y	Y	low	Y	Y	low	Y	Y	Y	Y	low
Wåhlander 2002	unclear	unclear	Y	Y	unclear	Y	Y	low	Y	Y	low	Y	Y	Y	Y	low
Walpoth 1998	unclear	unclear	Y	Y	unclear	Y	Y	low	Y	Y	low	Y	Y	Y	unclear	unclear
Wang 1996	high	N	Y	Y	high	Y	Y	low	Y	Y	low	Y	Y	Y	Y	low

Meta-Regression eTables

eTable 1 – Acute Rejection, Adult, No Temporal Exclusion Criteria, Dichotomous

Covariate	k	Regression coefficient – magnitude of effect size modification	p-value
Conventional vs High Sensitivity Troponin / ISHLT 1990 vs 2004	8	0.2097	0.0006
Study Year	8	0.0142	0.0010
Number of Study Centres	8	0.1894	0.0154
Study Design	8	0.2227	0.0634
Mean Age	5	0.0414	0.1020
ISHLT Threshold for rejection (ISHLT 1990 2/ISHLT 2004 1R vs ISHLT 1990 3A/ISHLT 2004 2R)	8	0.1474	0.2631
Percentage Male	6	-0.6839	0.5260
Overall Risk of Bias – unclear (reference: low)	8	0.0847	0.5307
Troponin Threshold	8	-0.0407	0.6290
Number of Patients	8	-0.0005	0.6382
SD Age	5	0.0096	0.6600
Troponin Subtype (I vs T)	8	-0.0397	0.6739
Overall Risk of Bias – high (reference: low)	8	-0.0186	0.8713
Number of Samples	7	0.0000	0.9698
Percentage Samples with Significant Rejection	8	0.0000	0.9922

eTable 1: Meta-regression table of dichotomous effect measures for acute cellular rejection in adults, with no exclusion of measurements from the early postoperative period. The Bayesian area under the receiver operating characteristic curve (BAUC) is the dependent variable for regression. k = number of studies reporting each covariate included in the analysis.

eTable 2 – Acute Rejection, Adult, No Temporal Exclusion Criteria, Continuous

Covariate	k	Regression coefficient – magnitude of effect size modification	p-value	95% CI (lb, ub)	Heterogeneity accounted for	Residual Heterogeneity (I^2)
SD Age	9	0.2739	0.0048	0.0836<br>0.4642	49.47%	94.13%
Study Year	11	0.1305	0.0224	0.0185<br>0.2425	33.85%	94.67%
Conventional vs High Sensitivity Troponin / ISHLT 1990 vs 2004 (reference: high sensitivity troponin / ISHLT 2004)	11	-1.8030	0.0307	-3.4379<br>-0.1680	29.06%	94.88%
Troponin Threshold	6	-4.6176	0.0418	-9.0628<br>-0.1723	40.31%	93.78%
Number of Study Centres	11	1.6736	0.0810	-0.2065<br>3.5537	18.01%	95.50%
Number of Patients	11	0.0209	0.1790	-0.0096<br>0.0514	6.75%	96.18%
Study Design (reference: prospective)	11	1.2622	0.2270	-0.7855<br>3.3099	5.56%	96.02%
Percentage Samples with Significant Rejection	11	0.0340	0.3990	-0.0450<br>0.1129	0.00%	96.51%
Number of Samples	10	0.0015	0.6399	-0.0048<br>0.0078	0.00%	96.86%
Percentage Male	9	-4.3464	0.6880	-25.5586<br>16.8658	0.00%	97.20%
Troponin Subtype (reference: troponin I)	11	0.3486	0.7380	-1.6942<br>2.3915	0.00%	96.66%
ISHLT Threshold for rejection (reference: ISHLT 1990 2/ISHLT 2004 1R)	11	0.1556	0.8900	-2.0499<br>2.3611	0.00%	96.89%
Mean Age	9	-0.0208	0.9340	-0.5133<br>0.4716	0.00%	97.25%
Overall Risk of Bias – unclear (reference: low)	11	0.8570	0.5535	-1.9781<br>3.6922	0.00%	96.78%
Overall Risk of Bias – high (reference: low)	11	1.4156	0.3418	-1.5032<br>4.3344		

eTable 2: Meta-regression table of continuous effect measures for acute cellular rejection in adults, with no exclusion of measurements from the early postoperative period. The standardised mean difference (SMD) is the dependent variable for regression. ‘k’ is the

number of studies reporting each covariate included in the analysis. ‘Heterogeneity accounted for’ answers the question of how much of the difference in the true effect size in a meta-regression is due to the covariate. It is analogous to the R^2 statistic, which explains how much the variance of one variable explains the variance in another. ‘Residual heterogeneity (I^2)’ answers the question of how much of the between study heterogeneity is due to the covariate (the ‘residual’ heterogeneity), after removing the effect of random noise (sampling variability).

eTable 3 – Acute Rejection, Adult, Early Postoperative Exclusion Criteria, Dichotomous

Covariate	k	Regression coefficient – magnitude of effect size modification	p-value
Conventional vs High Sensitivity Troponin / Troponin Subtype (I vs T) / ISHLT 1990 vs 2004	8	0.2574	0.0270
Study Design	8	0.1729	0.0313
Study Year	8	0.0137	0.0548
Troponin Threshold	7	-1.4117	0.1082
Number of Patients	8	-0.0010	0.3340
ISHLT Threshold for rejection (ISHLT 1990 2/ISHLT 2004 1R vs ISHLT 1990 3A/ISHLT 2004 2R)	8	0.1297	0.3456
Overall Risk of Bias – unclear (reference: low)	8	0.1033	0.4660
Overall Risk of Bias – high (reference: low)	8	-0.0886	0.4701
Percentage Male	6	-0.4471	0.5070
Number of Samples	8	-0.0001	0.6249
SD Age	5	0.0086	0.7770
Percentage Samples with Significant Rejection	8	-0.0011	0.8330
Mean Age	5	-0.0007	0.9350
Months excluded post transplantation	8	0.0021	0.9477
Number of Study Centres	8	N/A as all single centre	

eTable 3: Meta-regression table of dichotomous effect measures for acute cellular rejection in adults, with exclusion of measurements from the early postoperative period. The Bayesian area under the receiver operating characteristic curve (BAUC) is the dependent variable for regression. k = number of studies reporting each covariate included in the analysis.

eFigures

eFigure 1

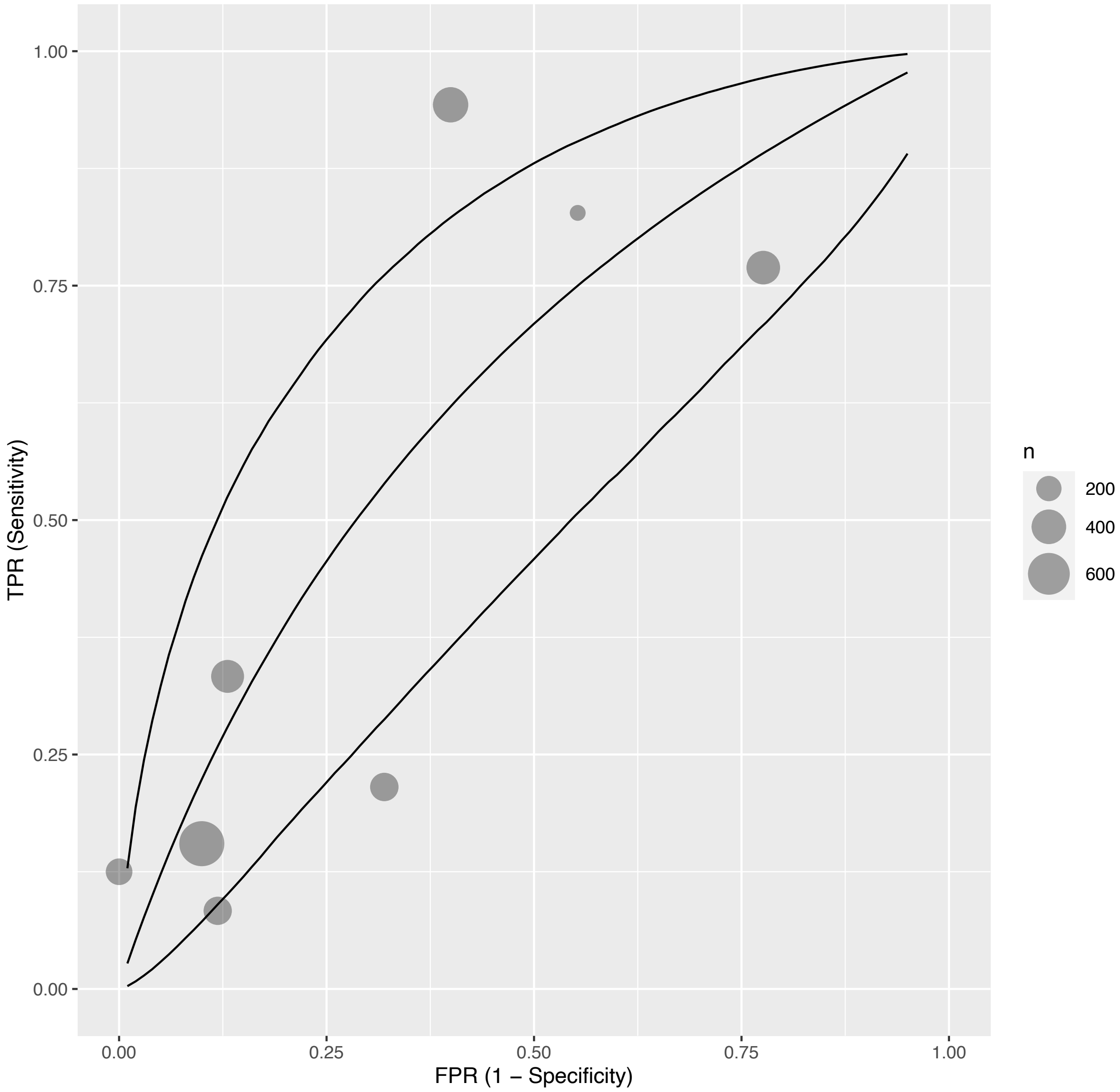

eFigure 1: Bayesian summary receiver operating characteristic curve showing summary diagnostic accuracy of recipient troponin in acute rejection with no temporal exclusions, with upper and lower 95% credible bands – sensitivity analysis using troponin T instead of troponin I for Wang 1996 [11].

eFigure 2

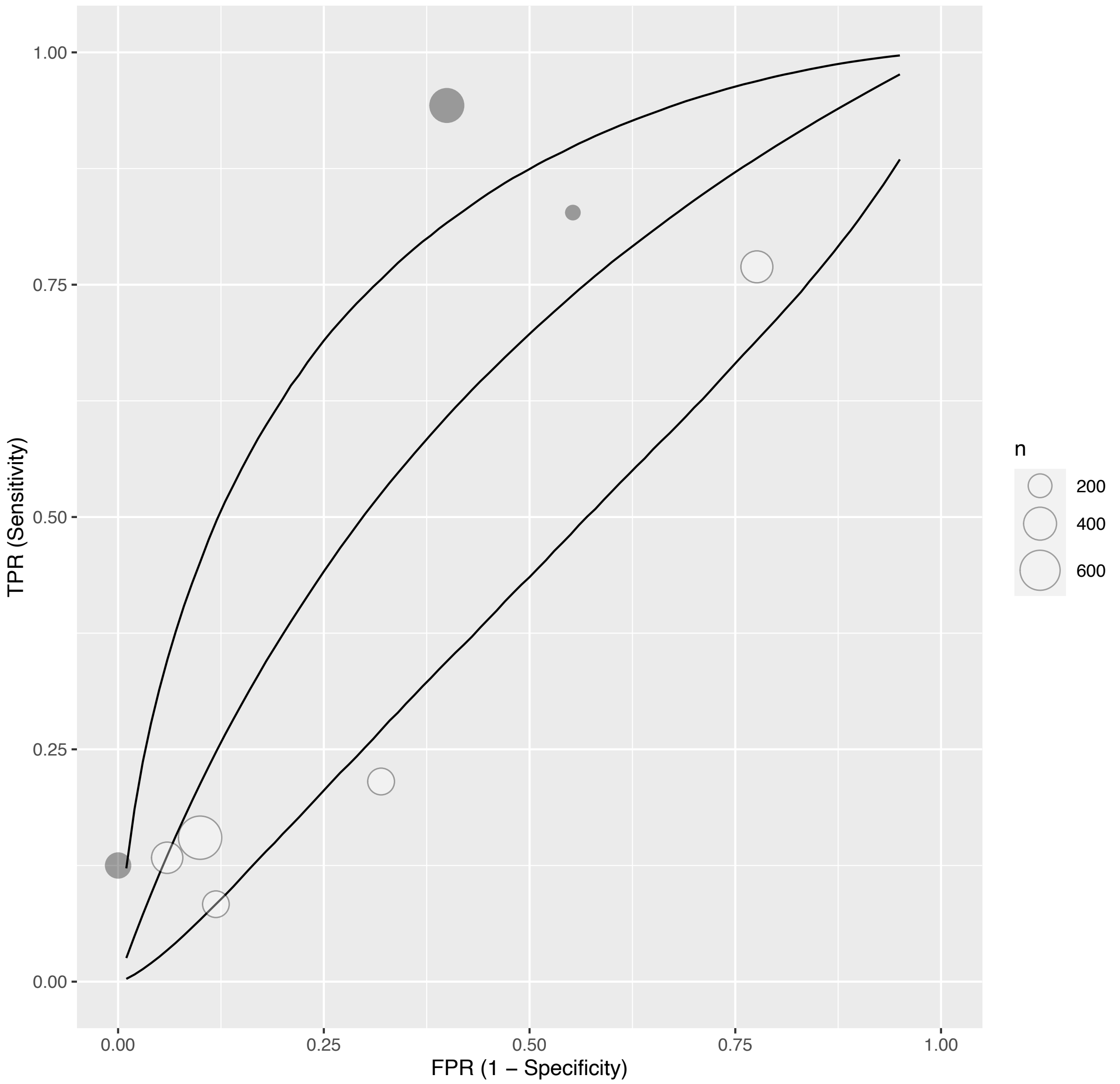

eFigure 2: Bayesian summary receiver operating characteristic curve showing summary diagnostic accuracy of recipient troponin in acute rejection with no temporal exclusions, with upper and lower 95% credible bands – subgroup analysis with International Society for Heart and Lung Transplantation (ISHLT) 2004 and high sensitivity troponin assays (black) vs ISHLT 1990 and conventional troponin assays (white).

eFigure 3

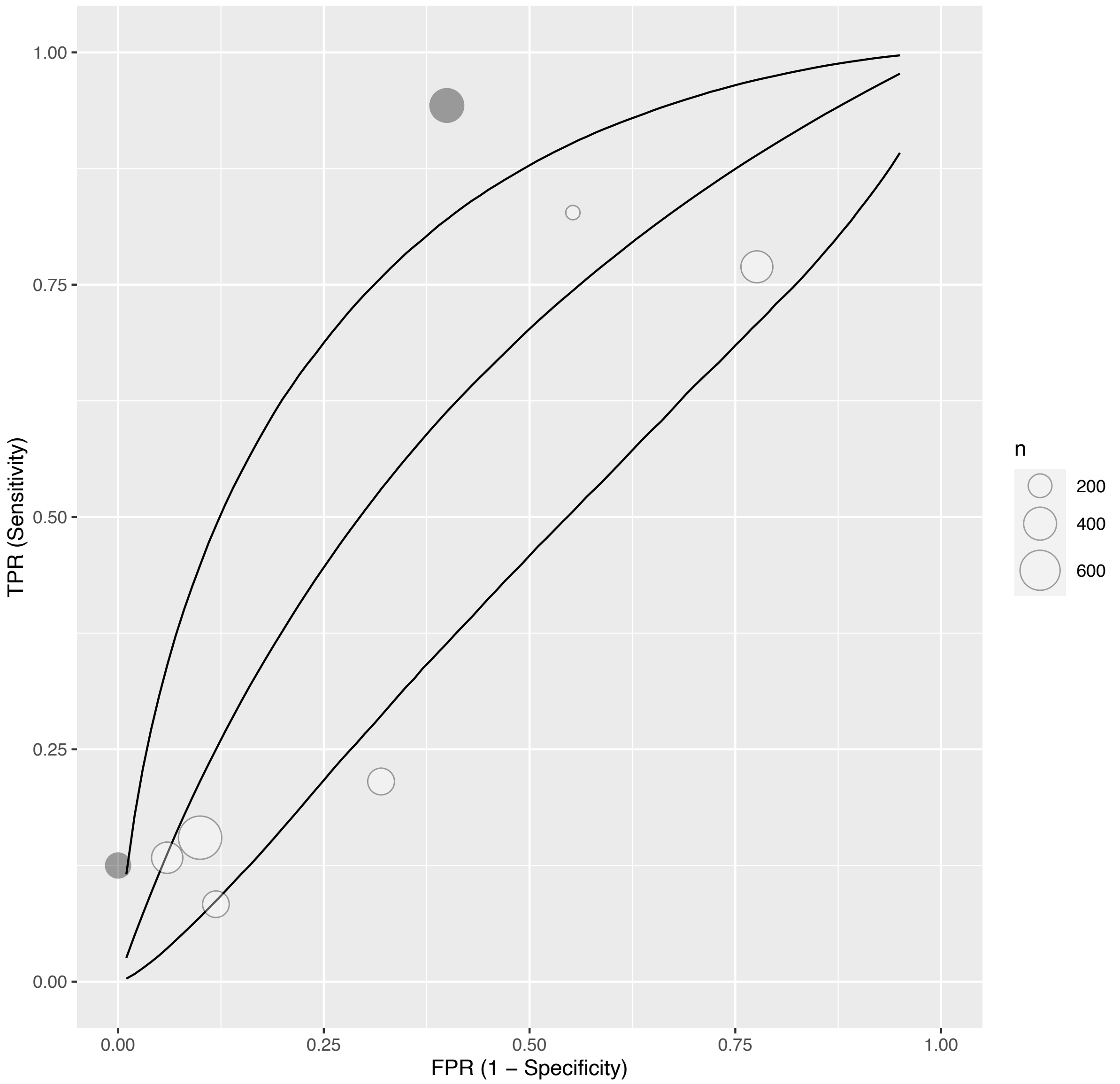

eFigure 3: Bayesian summary receiver operating characteristic curve showing summary diagnostic accuracy of recipient troponin in acute rejection with no temporal exclusions, with upper and lower 95% credible bands – subgroup analysis with multicentre (black) vs single centre (white) study designs.

eFigure 4

Study or Subgroup	Acute Rejection Mean	SD	Total	No Rejection Mean	SD	Total	Weight	Std. Mean Difference IV, Random, 95% CI
Ahn 2015	0.0662	0.1428	18	0.0163	0.0207	234	9.5%	1.18 [0.69, 1.67]
Cauliez 2000	0.1	0.0001	9	0.2478	0.1535	91	9.2%	−1.00 [−1.70, −0.30]
Forni 2000	0.094	0.083	13	0.1255	0.1199	372	9.4%	−0.26 [−0.82, 0.29]
Halwachs 1996	0.4585	0.0185	2	0.5453	0.6845	181	7.9%	−0.13 [−1.52, 1.27]
Hossein-Nia 1996	0.015	0.025	8	0.036	0.073	206	9.2%	−0.29 [−1.00, 0.42]
Hsu 2003	19.2275	16.7762	16	0.6525	0.6473	55	9.2%	2.34 [1.66, 3.03]
Mendez 2014	0.164	0.0132	16	0.074	0.0534	208	9.4%	1.74 [1.20, 2.27]
Mullen 2002	0.02	0.02	2	0.08	0.01	171	7.6%	−5.92 [−7.45, −4.39]
Munoz-Esparza 2011	0.425	0.8109	29	0.0867	0.124	38	9.5%	0.62 [0.12, 1.11]
Ogawa 2005	0.1149	0.1094	24	0.081	0.0798	659	9.5%	0.42 [0.01, 0.83]
Patel 2014	0.2233	0.2149	35	0.0341	0.0238	383	9.6%	2.88 [2.48, 3.28]
**Total (95% CI)**			**172**			**2598**	**100.0%**	**0.26 [−0.64, 1.16]**

Heterogeneity: $Tau^2 = 2.19$; $Chi^2 = 263.78$, df = 10 (P < 0.00001); $I^2 = 96\%$
Test for overall effect: Z = 0.57 (P = 0.57)

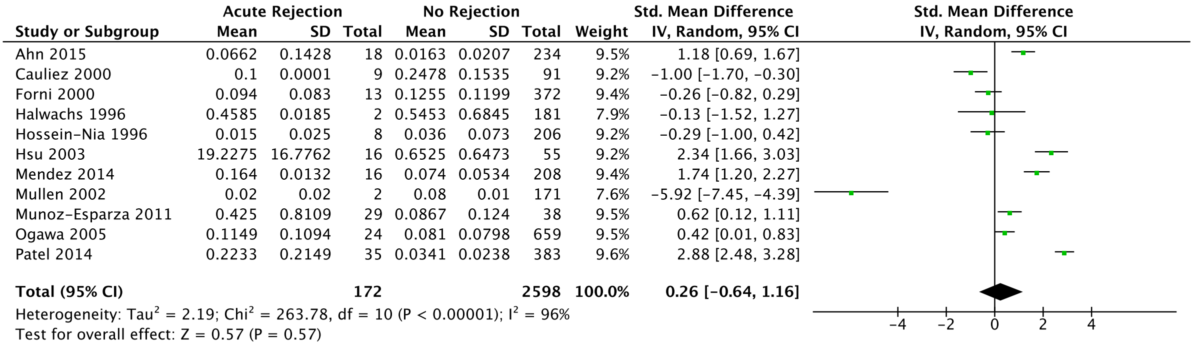

eFigure 4: Forest plot of standardised mean differences for elevated recipient troponin in diagnosing acute rejection post cardiac transplantation, with no temporal exclusions – sensitivity analysis using troponin T instead of troponin I for Mullen 2002 [14].

eFigure 5

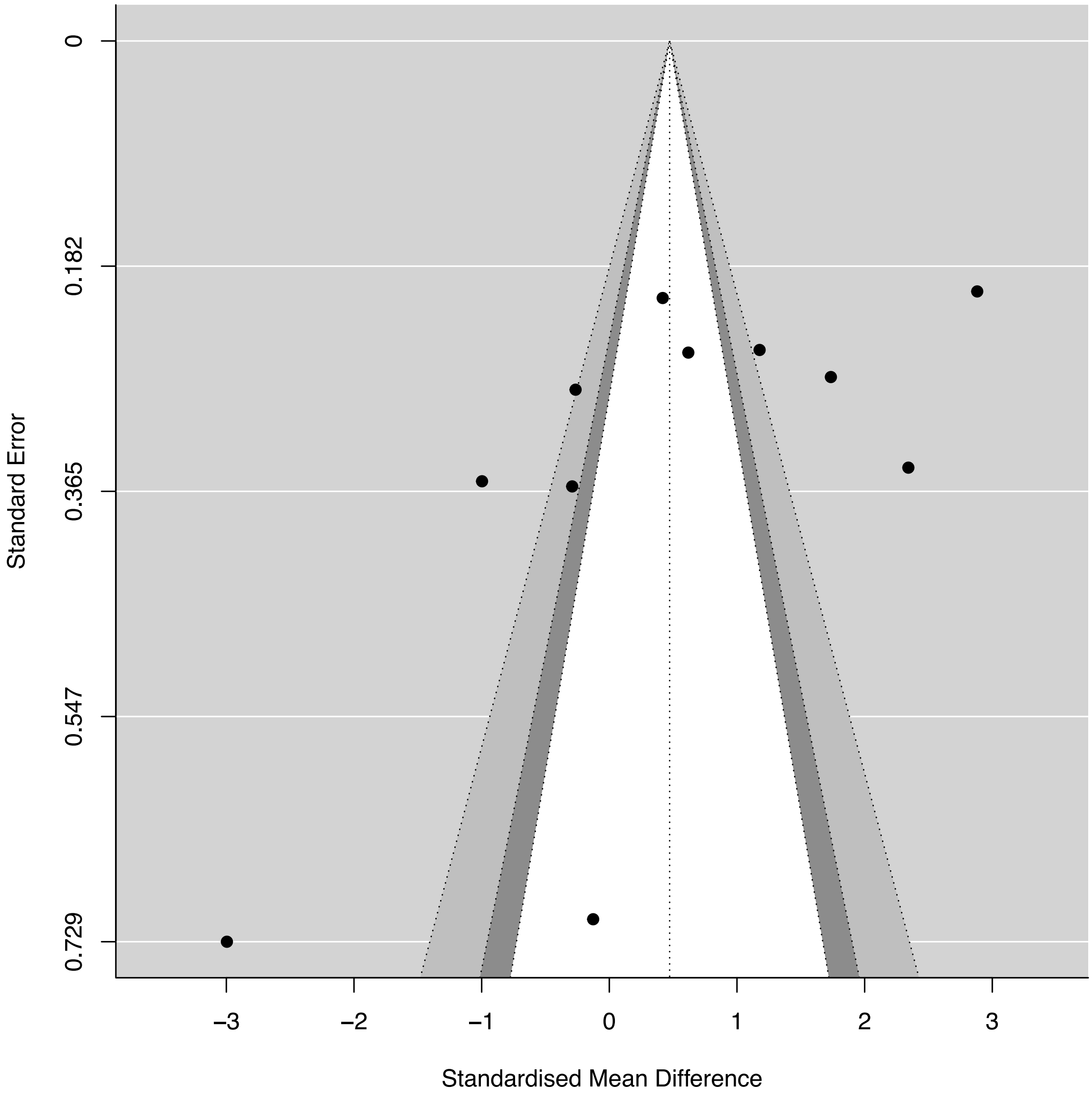

eFigure 5: Funnel plot for estimation of publication bias for elevated recipient troponin in diagnosing acute rejection post cardiac transplantation, with no temporal exclusions.

eFigure 6

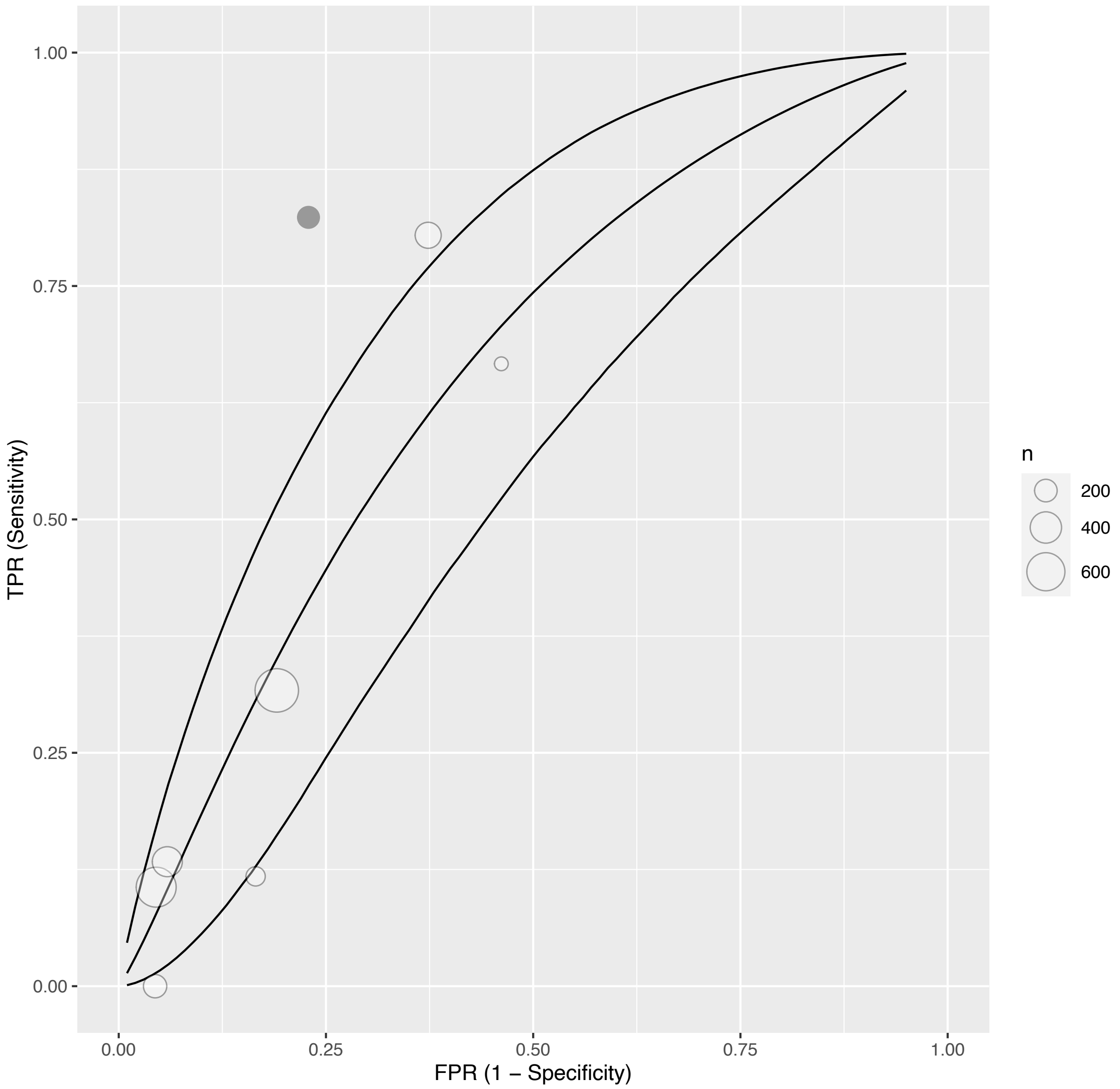

eFigure 6: Bayesian summary receiver operating characteristic curve showing summary diagnostic accuracy of recipient troponin in acute rejection with early postoperative measurements excluded, with upper and lower 95% credible bands – subgroup analysis with ISHLT 2004, high sensitivity troponin I (black) vs ISHLT 1990, conventional troponin T (white).

Supplement References

1. Ahn KT, Choi JO, Lee GY, Park HD, Jeon ES. Usefulness of high-sensitivity troponin I for the monitoring of subclinical acute cellular rejection after cardiac transplantation. *Transplant Proc*. 2015; **47**: 504-10.
2. Alexis JD, Lao CD, Selter JG, et al. Cardiac troponin T: a noninvasive marker for heart transplant rejection? *J Heart Lung Transplant*. 1998; **17**: 395-8.
3. Balduini A, Campana C, Ceresa M, et al. Utility of biochemical markers in the follow-up of heart transplant recipients. *Transplant Proc*. 2003; **35**: 3075-8.
4. Chance JJ, Segal JB, Wallerson G, et al. Cardiac troponin T and C-reactive protein as markers of acute cardiac allograft rejection. *Clin Chim Acta*. 2001; **312**: 31-9.
5. Dengler TJ, Zimmermann R, Braun K, et al. Elevated serum concentrations of cardiac troponin T in acute allograft rejection after human heart transplantation. *J Am Coll Cardiol*. 1998; **32**: 405-12.
6. Dyer AK, Barnes AP, Fixler DE, et al. Use of a highly sensitive assay for cardiac troponin T and N-terminal pro-brain natriuretic peptide to diagnose acute rejection in pediatric cardiac transplant recipients. *Am Heart J*. 2012; **163**: 595-600.
7. Faulk WP, Labarrere CA, Torry RJ, Nelson DR. Serum cardiac troponin-T concentrations predict development of coronary artery disease in heart transplant patients. *Transplantation*. 1998; **66**: 1335-9.
8. Gleissner CA, Klingenberg R, Nottmeyer W, et al. Diagnostic efficiency of rejection monitoring after heart transplantation with cardiac troponin T is improved in specific patient subgroups. *Clin Transplant*. 2003; **17**: 284-91.
9. Vazquez-Rodriguez JM, Crespo-Leiro MG, Pampin-Conde MF, et al. Cardiac troponin T is not a marker of biopsy-proven cellular rejection. *J Heart Lung Transplant*. 1999; **18**: 172.
10. Wahlander H, Kjellstrom C, Holmgren D. Sustained elevated concentrations of cardiac troponin T during acute allograft rejection after heart transplantation in children. *Transplantation*. 2002; **74**: 1130-5.
11. Wang CW, Steinhubl SR, Castellani WJ, et al. Inability of serum myocyte death markers to predict acute cardiac allograft rejection. *Transplantation*. 1996; **62**: 1938-41.
12. Zimmermann R, Baki S, Dengler TJ, et al. Troponin T release after heart transplantation. *Br Heart J*. 1993; **69**: 395-8.
13. Verde PE. bamdit: An R Package for Bayesian Meta-Analysis of Diagnostic Test Data. *Journal of Statistical Software*. 2018; **86**: 1-32.
14. Mullen JC, Bentley MJ, Scherr KD, et al. Troponin T and I are not reliable markers of cardiac transplant rejection. *Eur J Cardiothorac Surg*. 2002; **22**: 233-7.
